# Aerodynamic Performance of a Passive Pitching Model on Bionic Flapping Wing Micro Air Vehicles

**DOI:** 10.1155/2019/1504310

**Published:** 2019-12-10

**Authors:** Jinjing Hao, Jianghao Wu, Yanlai Zhang

**Affiliations:** School of Transportation Science and Engineering, Beihang University, Beijing 100191, China

## Abstract

Reducing weight and increasing lift have been an important goal of using flapping wing micro air vehicles (FWMAVs). However, FWMAVs with mechanisms to limit the angle of attack (*α*) artificially by active force cannot meet specific requirements. This study applies a bioinspired model that passively imitates insects' pitching wings to resolve this problem. In this bionic passive pitching model, the wing root is equivalent to a torsional spring. *α* obtained by solving the coupled dynamic equation is similar to that of insects and exhibits a unique characteristic with two oscillated peaks during the middle of the upstroke/downstroke under the interaction of aerodynamic, torsional, and inertial moments. Excess rigidity or flexibility deteriorates the aerodynamic force and efficiency of the passive pitching wing. With appropriate torsional stiffness, passive pitching can maintain a high efficiency while enhancing the average lift by 10% than active pitching. This observation corresponds to a clear enhancement in instantaneous force and a more concentrated leading edge vortex. This phenomenon can be attributed to a vorticity moment whose component in the lift direction grows at a rapid speed. A novel bionic control strategy of this model is also proposed. Similar to the rest angle in insects, the rest angle of the model is adjusted to generate a yaw moment around the wing root without losing lift, which can assist to change the attitude and trajectory of a FWMAV during flight. These findings may guide us to deal with various conditions and requirements of FWMAV designs and applications.

## 1. Introduction

The requirements for the design of flapping wing micro air vehicles (FWMAVs) include excellent aerodynamic performance, high efficiency, and satisfactory maneuverability. However, balancing all these standards is difficult for existing FWMAVs. Fortunately, flying creatures have been considered as a basis for proposing new innovations related to flying. For example, insects can manipulate their wings to complete a series of complex movements, such as hovering, climbing, braking, accelerating, and turning. Inspired by these phenomena, researchers have attempted to adopt the physiological characteristics of insects and apply a bionic model to artificial FWMAVs. Researchers have also conducted a series of studies on this topic. For example, Ennos [1] stated that torsion is necessary to design insect wings because insects have to twist their wings between wingbeats to optimize the performance of an aerofoil. Nevertheless, the kinematic mechanism of insect wings is difficult to fully understand because of the complex structure of organisms. On the one hand, this scenario is a typical type of a fluid-structure coupling problem, and the interaction between wings and the unsteady flow field generated during their movement is highly complicated. On the other hand, the mechanism through which insects control their wings involves numerous muscle structures and neural activities but remains poorly understood. Beatus and Cohen [2, 3] summarized this intractable behavior by applying a reduced-order approach in which the wing hinge of insects and fluid-structure interactions are represented by simplified models. Then, a passive pitching model based on the torque exerted by insects on their wings was proposed. In this model, the wing root of an insect is equivalent to a torsional spring [4]. The pitching dynamics of wings are assumed to be passively determined by combining aerodynamic, torsional, and inertial moments. Bergou et al. [5] also confirmed that pitching is passive by showing that aerodynamic and inertial forces are sufficient to pitch a wing without the aid of muscles.

Numerous theories and experiments have shown that a passive pitching model is generally accepted. Ishihara et al. [6, 7] applied a novel fluid-structure interaction similarity law to two- and three-dimensional wings and analyzed the motion of a passive pitching wing through computational and experimental methods. They mainly discussed the contributions of a wing's elastic, aerodynamic, and inertial forces and tried to find the important control parameters of passive pitching motion. Chen et al. [8] successfully used this passive pitching model to estimate aerodynamic forces with quasisteady and numerical methods. They found that wings with stiff hinges achieve a favorable pitching kinematic that leads to large mean lift forces. This model is applicable not only to a hovering state but also to a maneuvering state. Beatus and Cohen [3] explained wing pitch modulation in maneuvering fruit flies by an interplay between aerodynamics and a torsional spring. Zeyghami et al. [9] studied the passive pitching of a flapping wing in turning flight and concluded that passive wing kinematic modulations are fast and energetically efficient. Similarly, our study equated the wing's flexibility to a torsional spring at the wing root located close to the leading edge. This study is mainly aimed at determining whether aerodynamic force and efficiency could be improved if we used this passive pitching model to design FWMAVs and identifying whether the maneuverability of FWMAVs would be compromised.

In this study, we investigate the aerodynamic performance of a FWMAV with a Reynolds number of 10^4^. A series of analyses is conducted on the basis of a bionic passive pitching model through a 3D numerical simulation and a systematic comparison among them. To develop a desirable outcome of a FWMAV design, we discuss the effect of several dominant parameters, such as torsional stiffness and rest angle of torsional spring, on aerodynamic performance. We find that a FWMAV with passive pitching wings more likely reduces weight, increases lift, and shows great potential for flight control.

## 2. Modeling and Method

### 2.1. Wing Model and Kinematics

**Figure 1:**
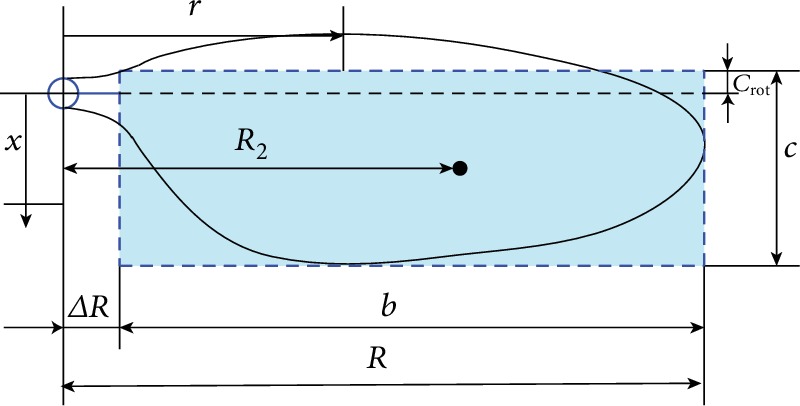
Geometric parameters of a flapping wing. *b* is the unilateral wingspan, *c* is the mean chord length, *c*_rot_ is the distance between the leading edge and the rotation axis, *R* is the radius of the wing tip, Δ*R* is the distance between the wing root and the flapping axis, and *R*_2_ is the radius of the second moment of the wing area.

Insect wings have a dynamic geometry. They are made of different materials and exhibit varying structures to adapt to different flight environments. In practical applications, artificial wings cannot achieve the same effect as insect wings. Consequently, simplifications are frequently adopted. In this study, we use a rectangle to approximate a planar shape and regard a flapping wing as a thin plate with a uniform density (Figure 1). The reason why the rectangular model wings are used is as follows. Luo and Sun [10] have investigated the effect of wing planform on the aerodynamic force production of model insect wings in rotating at Reynolds numbers 200 and 3500 at an angle of attack of 40° in 2005 and revealed that the variation in wing shape and aspect ratio (from 2.84 to 5.45) has minor effects on the lift and drag coefficients. Based on their conclusions, we neglected the effect of planar shape and focused on other important parameters such as torsional stiffness in this paper. Besides, the rectangular model wing has been extensively used in many numerical simulations [7, 11], which can be regarded as a typical case to illustrate a universal conclusion.

**Figure 2:**
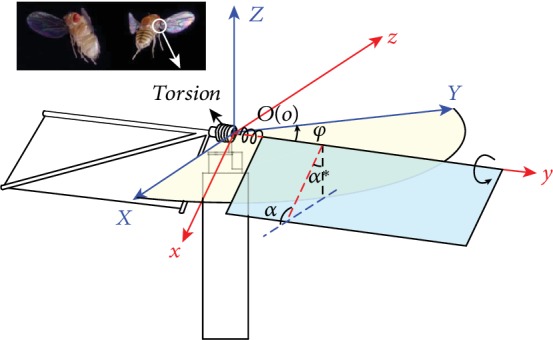
Bioinspired passive pitching model and coordinate system.

To clearly describe the 3D motion of a flapping wing and accurately analyze its force, we establish two coordinate systems with the same origin located on the wing root (Figure 2). The inertial system *O*‐*XYZ* is located on the ground, whereas the *OXY* plane is parallel to the horizontal plane. The *OX* axis is oriented toward the trailing edge, the *OZ* axis is opposite to the direction of gravity, and the *OY* axis is determined on the basis of the right-hand rule. The coordinate system *O*‐*xyz* is fixed on the wing. *Ox* and *Oy* axes are along the chordwise and spanwise directions, respectively. The *Oz* axis is determined on the basis of the right-hand rule.

Insects generally have three degrees of freedom while hovering. The motion perpendicular to the flapping plane is relatively small and frequently overlooked during simplification. Therefore, the motion of a wing can be approximately decomposed into flapping and pitching, which are described by the flapping angle *φ* and the angle of attack *α*, respectively. Flapping refers to the rotation around the *OZ* axis, whereas pitching corresponds to the rotation around the *Oy* axis.

The flapping motion can be described by a trigonometric function as follows:

(1) $$\begin{array}{c} \dot{\varphi }=\frac{\pi }{360}\Phi \sin\left(2\pi T\right), \end{array}$$

where *Φ* and *T* are the flapping amplitude and nondimensional time, respectively. Wing kinematic parameters are nondimensionalized. The mean chord length and the average velocity at the span location *R*_2_ are taken as the reference length *c* and the velocity *U*, respectively. *U* is defined as 2*Φfλc*/180, where *f* and *λ* are the flapping frequency and the wing aspect ratio, respectively. Reference time is defined as *c*/*U*, and the nondimensional time *T* is *t*/(*c*/*U*). These reference values are used to nondimensionalize wing kinematic parameters, forces, and moments in this study. Unless otherwise specified, the physical quantities in the following sections are in a dimensionless form.

In previous studies, the wing is thought to pitch in accordance with a preset form (e.g., sinusoidal curve and trapezoidal curve). In general, *α* takes a constant value except at the beginning or near the end of a half-stroke [12]. $\dot{\alpha }$ is given by

(2) $$\begin{array}{c} \dot{\alpha }=0.5\omega_{r}\left\{1-\cos\left[\frac{2\pi \left(t-t_{r}\right)}{\Delta \tau_{r}}\right]\right\},\;t_{r}\leq t\leq t_{r}+\Delta \tau_{r}, \end{array}$$

where **ω**_*r*_ is the mean angular velocity, *t*_*r*_ is the time at which the pitching motion starts, and Δ*τ*_*r*_ is the nondimensional time interval over which the rotation lasts. The constant *α* in the upstroke and downstroke are defined as *α*_*u*_ and *α*_*d*_, respectively. In the time interval of Δ*τ*_*r*_, the wing *α* changes from *α*_*u*_ to *α*_*d*_.

An active pitching model artificially decouples *φ* from *α*, which considerably simplifies the analysis and calculation processes. This model is also widely used in quasisteady estimations. However, this model also exhibits unavoidable drawbacks in the design and application of FWMAVs. It creates additional burdens to mechanisms and does not reflect actual pitching motion. Under this circumstance, a passive pitching model based on bionics becomes widely recognized. This model was first proposed because deformations play an important role on the aerodynamic performance of flapping wings, but it is difficult to directly simulate the deformation process as a result of the interaction between flexible wing with the surrounding flow and the complex structure of the insect wing. In this paper, we considered the effect of deformation with a reduced-order approach [3]. For most dipteran insects, the narrow root region of wings is flexible, thereby allowing them to rotate around the axis in the leading edge [6]. On the basis of this structural feature, we compress the torsional flexibility of a flapping wing to the wing root and simulate it with a torsional spring [5]. The variation in *α* can be obtained as follows.

In a passive pitching model, *α* is determined in accordance with the coupled dynamic equations of aerodynamic and elastic forces. A flapping wing is considered as a rigid plate, and the moment generated by the torsional spring at a rotating axis can be expressed as

(3) $$\begin{array}{c} M_{\text{torsion}}=-k\left(\alpha -\alpha_{0}\right), \end{array}$$

where *k* and *α*_0_ are the elastic coefficient and rest angle of the torsional spring, respectively.

The initial state of a flapping wing can be artificially specified. In our study, it is set perpendicular to the *OXY* plane (*α*_0_ = 90°). When the wing begins to flap, the aerodynamic force is substantially perpendicular to the wing surface, thereby generating a moment around the wing leading edge and causing the wing to rotate. At this time, the torsional spring applies a moment opposite to the aerodynamic moment. Thus, the two moments interact with the inertial moment and reach equilibrium. In comparison with the aerodynamic force, the weight of the wing is essentially negligible because it is typically less than 0.5% of the entire weight [13]. The aerodynamic and torsional spring moments increase as the average flapping speed increases, resulting in a large pitch angle.

The coordinate system fixed on the wing rotates at an angular velocity $\dot{\varphi }$ during motion. Thus, the transformation relationship between coordinates *O*‐*XYZ* and *O*‐*xyz* must be considered when the equation of *α* is derived:

(4) $$\begin{array}{c} \sum \tau ={\left(\frac{{dL}_{w}}{dt}\right)}_{OXYZ}={\left(\frac{{dL}_{w}}{dt}\right)}_{oxyz}+\omega \times L_{w}, \end{array}$$

where ∑*τ* is the external moment, *L*_*w*_ is the momentum moment of the wing relative to the origin of the coordinate system, and **ω** is the angular velocity of the wing.

In the coordinate *O*‐*xyz*, the projection of angular velocity in three directions can be expressed as

(5) $$\begin{array}{c} \left(\begin{array}{c} p\\ q\\ r \end{array}\right)=\left(\begin{array}{c} \omega_{x}\\ \omega_{y}\\ \omega_{z} \end{array}\right)=\left(\begin{array}{c} 0\\ \dot{\alpha }\\ 0 \end{array}\right)+\left(\begin{array}{ccc} \cos\alpha & 0 & \sin\alpha \\ 0 & 1 & 0\\ -\sin\alpha & 0 & \cos\alpha \end{array}\right)\left(\begin{array}{c} 0\\ 0\\ \dot{\varphi } \end{array}\right)=\left(\begin{array}{c} \dot{\varphi }\sin\alpha \\ \dot{\alpha }\\ \dot{\varphi }\cos\alpha \end{array}\right). \end{array}$$

The component form of the dynamic equation can be expressed as follows:

(6) $$\begin{array}{c} \left\{\begin{array}{c} I_{xx}\frac{dp}{dt}+\left(I_{yy}-I_{zz}\right)qr-I_{xy}\left(pr+\frac{dq}{dt}\right)=\tau_{x},\\ I_{yy}\frac{dq}{dt}+\left(I_{zz}-I_{xx}\right)pr+I_{xy}\left(qr-\frac{dp}{dt}\right)=\tau_{y},\\ I_{zz}\frac{dr}{dt}+\left(I_{xx}-I_{yy}\right)pq+I_{xy}\left(p^{2}-q^{2}\right)=\tau_{z}, \end{array}\right. \end{array}$$

where *τ*_*x*_, *τ*_*y*_, and *τ*_*z*_ are the components of the external moment in the directions *ox*, *oy*, and *oz*, respectively. The moment of the inertia of the wing to different axes and the inertial product can be expressed as

(7) $$\begin{array}{c} I_{xx}=\int \left(y^{2}+z^{2}\right)dm,\\ I_{yy}=\int \left(x^{2}+z^{2}\right)dm,\\ I_{zz}=\int \left(x^{2}+y^{2}\right)dm,\\ I_{xy}=\int xydm,\\ I_{yz}=\int yzdm,\\ I_{xz}=\int xzdm. \end{array}$$

When the wing is regarded as a flat plate and placed on the *Oxy* plane, the wing is thin and can be disregarded. Thus, *z* = 0. The preceding equation can be simplified as

(8) $$\begin{array}{c} I_{xz}=I_{yz}=0,\\ I_{zz}=I_{xx}+I_{yy}. \end{array}$$

An elastic restoring torque, which acts on the rotating axis of the wing, is generated when the torsional spring is deformed by an external force. Therefore, only the spanwise direction should be considered:

(9) $$\begin{array}{c} M_{\text{aero}}-k\left(\alpha -\alpha_{0}\right)=I_{yy}\left(\ddot{\alpha }+pr\right)-I_{xy}\left(\dot{p}-qr\right). \end{array}$$

Finally, the equation of *α* can be written as

(10) $$\begin{array}{c} \ddot{\alpha }=\frac{M_{\text{aero}}-k\left(\alpha -\alpha_{0}\right)}{I_{yy}}+\frac{I_{xy}}{I_{yy}}\ddot{\varphi }\sin\alpha -{\dot{\varphi }}^{2}\sin\alpha \cos\alpha , \end{array}$$

where *M*_aero_ is the aerodynamic moment acting on the wing. This equation is solved using the improved Euler scheme, and *α* is computed from the time integration.

### 2.2. Governing Equations and Solution Method

The governing equations of the flow are 3D incompressible unsteady Navier-Stokes equations, which are written in the coordinate system *O*‐*XYZ* in the following dimensionless form [14]:

(11) ∇·u=0,∂u∂t+u·∇u+∇p−1Re∇2u=0,

where **u** is the velocity vector and *p* is the static pressure. Re is defined as *Uc*/*υ*, where *υ* is the kinematic viscosity of the fluid. The governing equations are solved using a pseudocompressibility method based on the upwind scheme [15, 16]. We introduce a partial derivative term of pressure versus pseudotime in the continuous equation and transform the elliptic continuous equation into a hyperbolic continuous equation. Thus, the dimensionless flow control equation is transformed into a hyperbolic equation, which considerably improves the efficiency of the solution. We verified the numerical solution method in our past relevant research, and our previous conclusions are directly used in the present work [12, 14, 17–19].

Once the Navier-Stokes equations are numerically solved, the fluid velocity components and pressure at discretized grid points for each time step are available. The aerodynamic forces acting on the wing are calculated from the pressure and the viscous stress on the wing surface [14]. The force and moment coefficients are computed by

(12) $$\begin{array}{c} C_{F}=\frac{F}{1/2\rho U^{2}S},\\ C_{M}=\frac{M}{1/2\rho U^{2}Sc}, \end{array}$$

where *ρ* is the fluid density and *S* is the wing area. The component of *C*_*F*_ in the *OZ* direction is the lift coefficient *C*_*L*_. The aerodynamic power coefficient *C*_*P*_ is given as *C*_*p*_ = *C*_*M*_ · **ω**, where **ω** is the angular velocity vector in the coordinate system *O*‐*XYZ*. The average lift coefficient $\bar{C_{L}}$ and the aerodynamic power coefficient $\bar{C_{P}}$ are computed by averaging *C*_*L*_ and *C*_*P*_ in a flapping period, respectively. Aerodynamic efficiency *η*, which measures the wing aerodynamic power consumption to produce a certain amount of lift, is defined as

(13) $$\begin{array}{c} \eta =\frac{{\bar{C_{L}}}^{3/2}}{\bar{C_{P}}}. \end{array}$$

As a result of interaction between flapping wing and its own steady flow, the equation of *α* (equation (10)) and the Navier-Stokes equations (equation (11)) are coupled in the solution process. In order to solve this coupled dynamic problem, we refer to the Euler predictor-corrector method. Supposing that *α* of the wing is known at a certain time step, the boundary condition of the Navier-Stokes equations can be known and the flow equations can be solved to provide the aerodynamic forces and moments at this time step. Then, the value of *α* would be updated and the equations of motion would be marched to the next time step. This process is repeated in the following time steps. In theory, the iteration needs to be continued at a certain time step until the aerodynamic moments and *α* of the wing no longer change. But Wu et al. confirmed that the Euler predictor-corrector method has sufficient accuracy in practical application [20].

### 2.3. Validation

**Figure 3:**
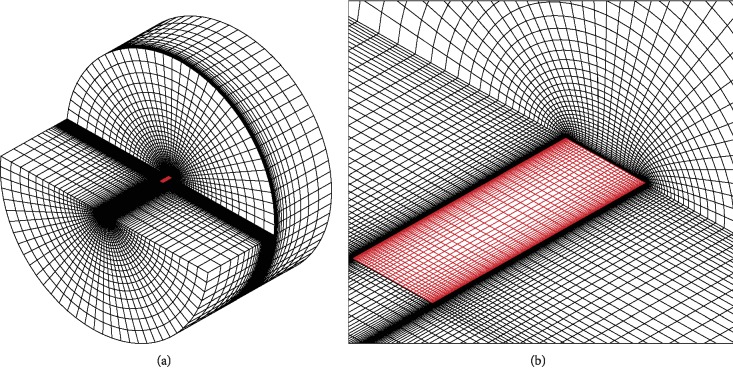
(a) Complete grid and (b) surface mesh.

The velocity and the pressure in the flow field around the wing are obtained using an O-H grid (Figure 3). A typical case is selected and tested in which the domain parameters are as follows: Re = 16100, *λ* = 3, *Φ* = 120°, and *T* = 7.255.

The Reynolds number of most insects and flapping creatures generally lies within the range of 10^2^~10^3^ because of their small size and weight. For example, the Reynolds number of *Drosophila* is approximately 160, its total weight is less than 20 mg, and its wing length is only approximately 2.5 mm. For a bumblebee, these parameters are 1100, 175 mg, and 13 mm, respectively. In this study, we aim to design FWMAVs with a good load capacity in which the Reynolds number is slightly larger and reaches 10^4^. However, a laminar flow transition problem may occur under this scenario. Isogai et al. [21] compared the calculation results of laminar and turbulent flows to investigate issues related to flapping thrust and propulsion efficiency. They determined that the difference between the results is small when the reduced frequency is large. Moreover, no evident flow separation is observed, and the flow structure is similar to laminar and turbulent flows with only slight differences in several details. On the basis of the results of Isogai et al., we use laminar flow without introducing a turbulence model under a Reynolds number of 10^4^ in our calculation because the reduced frequency of our aircraft is within their conclusions.

**Figure 4:**
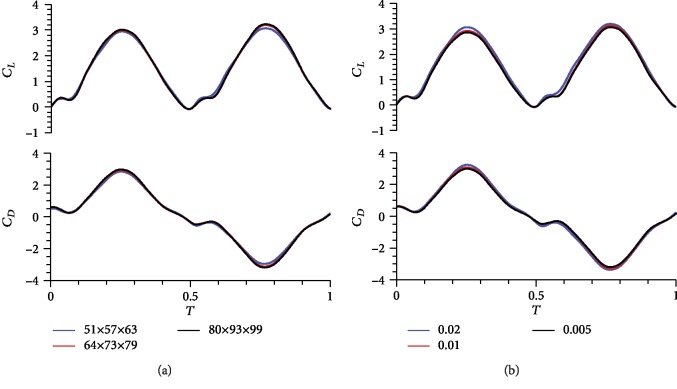
Comparison of three grids with different (a) densities and (b) time steps.

In numerical solutions, results and efficiency are affected by grid quality. As such, an appropriate grid density, a computational domain size, and a step value should be determined to ensure the accuracy and speed of calculation. Three sets of grids are evaluated to select the appropriate grid density: (a) 51 × 57 × 63 (around the wing section, in the normal direction of the wing surface, and in the spanwise direction of the wing), (b) 64 × 73 × 79, and (c) 80 × 93 × 99. These sets differ in density but have the same domain size of 40 times the chord length and a nondimensional time step value of 0.02. The time course of the aerodynamic force coefficients (*C*_*L*_ and *C*_*D*_) in one cycle is shown in Figure 4, indicating that the relatively coarse grid exhibits a remarkable deviation at the peak. The other parts of the three grids present good agreement.

Similarly, grids with different time step values are verified. A grid with a density of 64 × 73 × 79, a domain size of 40*c*, and a step value of 0.01 is selected to balance the calculation accuracy and the time cost.

## 3. Results and Discussions

The cases under typical conditions are chosen first to ensure comparability of the active and passive pitching wings: Re = 16100, *λ* = 3, *Φ* = 120°, and *T* = 7.255. *α* is an important parameter that influences the wing aerodynamic performance, so it is set to be changeable in this study. For the active pitching wing, *α*_*u*_ and *α*_*d*_ increase or decrease by 1.5 times on the basis of 45°. For the passive pitching wing, *k* increases or decreases by 8 times on the basis of 1.2, indirectly leading to the change in *α*.

### 3.1. Instantaneous *α* of the Passive Pitching Flapping Wing

**Figure 5:**
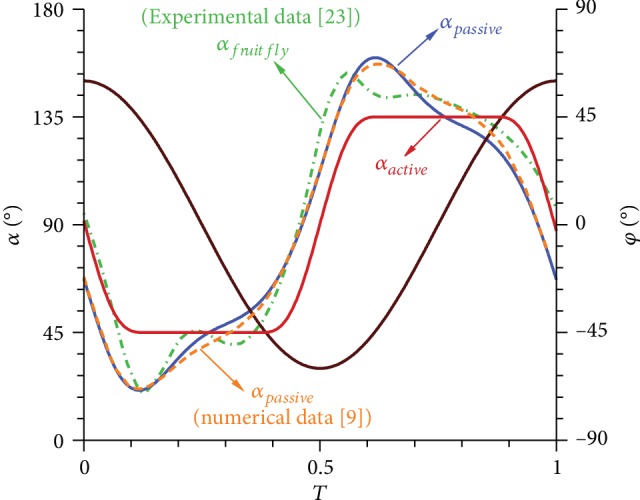
Curve of *α* from the active pitching model and the passive pitching model.

Studies on the mechanism of insect motion have shown that passive pitching is common during flight. A typical characteristic of *α* is “double peak oscillation” [11]. In particular, *α*^∗^ continues to increase during the first quarter of a wingbeat cycle and then gradually reaches the maximum value, where the first peak occurs. Subsequently, *α*^∗^ starts to decrease and rebounds slightly near the end of upstroke/downstroke, where the second peak occurs. Lastly, *α*^∗^ continues to decline and returns to its initial value. In Figure 5, the solution for the coupled dynamic equation corresponding to the simplified passive pitching model is similar to experimental results [22, 23] and computational results [9] listed in the previous literature, which exhibits a tendency quite different from the active pitching.

**Figure 6:**
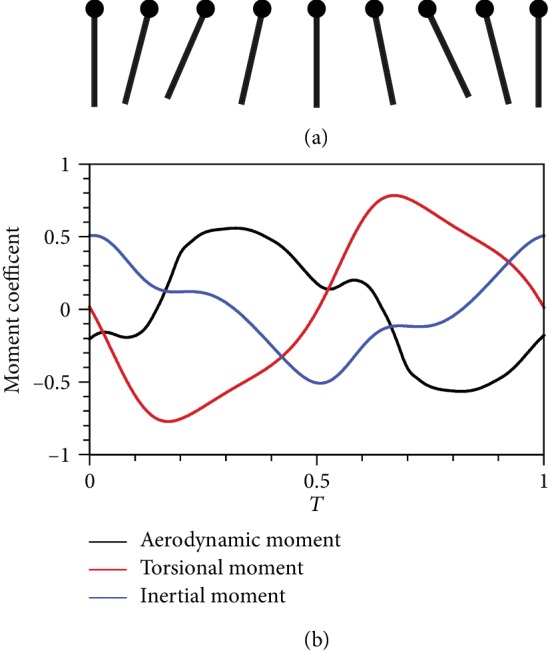
(a) Angle of attack and (b) three dimensionless moments of the passive pitching wing in one cycle.

To investigate the reason why the curve of *α* has two peaks, we analyze the variations in aerodynamic, torsional, and inertial moments within a wingbeat cycle to determine their interaction. Given that *α* changes continuously during flapping, a flapping wing has a positive pitching angular velocity, although it is in equilibrium at the beginning of upstroke (Figure 6). Initially, the effect of the inertial moment is stronger than those of aerodynamic and torsional moments. This condition causes the wing to move farther from the initial position, and *α*^∗^ increases continuously until it reaches the peak. Then, the effect of the inertial moment declines, whereas the effect of the torsional moment becomes considerable. As such, the flapping wing slowly returns to its initial position, which causes *α*^∗^ to decline. However, an exception occurs when the magnitude of the aerodynamic moment is the largest. The tendency of the wing to restore equilibrium is hindered, and *α*^∗^ increases slightly. Thus, another small peak can be observed in the curve. Subsequently, inertial moment prevails, thereby causing *α*^∗^ to decrease rapidly to the initial value. The situation in downstroke is similar.

### 3.2. Effect of Torsional Stiffness on the Aerodynamic Performance of the Passive Pitching Model

In the passive pitching model, *k* is an important parameter that considerably affects aerodynamic force and power consumption. Excess rigidity or flexibility deteriorates the performance. From Table 1, we can see that the torsional spring generates considerable elastic recovery moments when *k* is excessively large; i.e., the flapping wing is too rigid. Torsional moment offsets the effect of the aerodynamic moment within a short period each time the flapping wing rotates. Thus, the wing can only oscillate near the initial *α*. Although this condition can produce a certain amount of lift, it can also lead to a distinct increase in drag, thereby causing aerodynamic power consumption to become extremely high. Consequently, the overall aerodynamic efficiency is low. If *k* is excessively small, i.e., the flapping wing is too flexible, then the aerodynamic moment is clearly dominant. Once the wing starts to flap, *α* rapidly increases, and the wing becomes parallel to the inflow direction. The effect of torsional moment is weak and unable to maintain a stable periodic motion. Although drag and aerodynamic power are small, lift is considerably lower than the required value.

**Figure 7:**
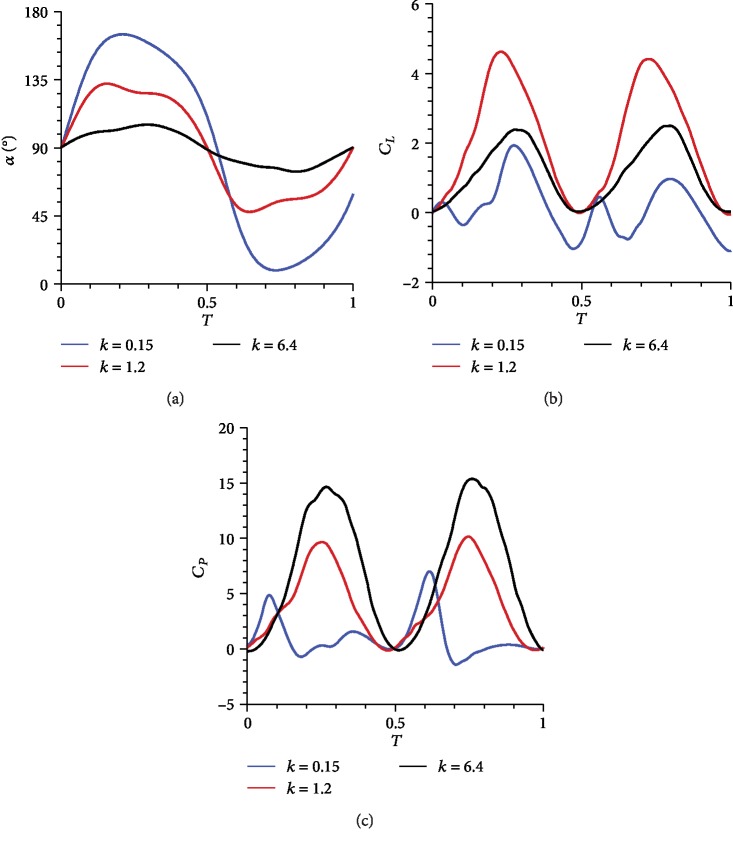
Instantaneous (a) *α*, (b) *C*_*L*_, and (c) *C*_*P*_ under different *k*.

Figure 7(a) shows the time history of *α* for cases with different *k*. These curves have similar trends with that reported previously by Kolomenskiy et al. [24]. They changed the torsional stiffness to obtain the one that coincides best with the experiment measurement, proving that this kind of simplified passive pitching model successfully reproduces the main dynamical features of some insects.

**Figure 8:**
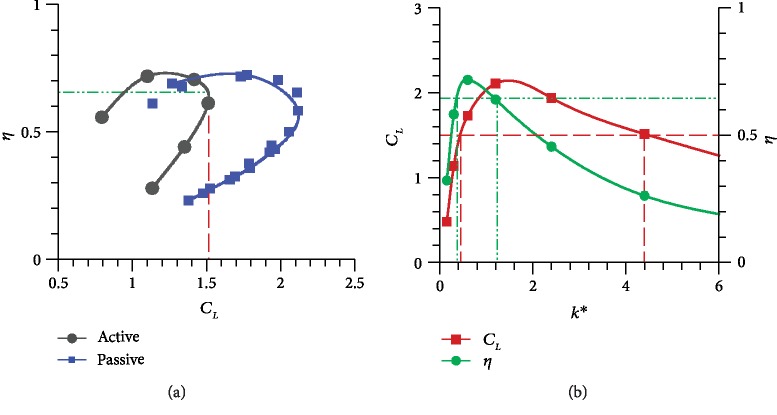
(a) Comparison between the two models of *η* versus $\bar{C_{L}}$. (b) $\bar{C_{L}}$ and $\bar{C_{P}}$ as a function of *k*.

The preceding analysis shows that a suitable *k* should be selected to design a FWMAV with good load capacity and high efficiency. Different values are taken at approximately equal intervals within the limitation of 0.15 ≤ *k* ≤ 6.4 to further explore the effect of this parameter on aerodynamic performance. For comparison, the related results of the active pitching wing are also plotted. The points of $\bar{C_{L}}$, $\bar{C_{P}}$, and *η* are fitted by the curves. The maximum $\bar{C_{L}}$ of the active pitching model is chosen as the baseline. The dashed line defines the lift constraint, and the points of the red curve above it represent the target lift that can be satisfied. Similarly, the dash dot line defines the aerodynamic efficiency constraint, and the points of the green curve above it indicate a higher aerodynamic efficiency. In Figure 8, the ideal range of *k* may be in the intersection of the two regions with an approximate value of 1–2.

### 3.3. Comparison of the Passive and Active Pitching Wing Aerodynamic Performance

**Figure 9:**
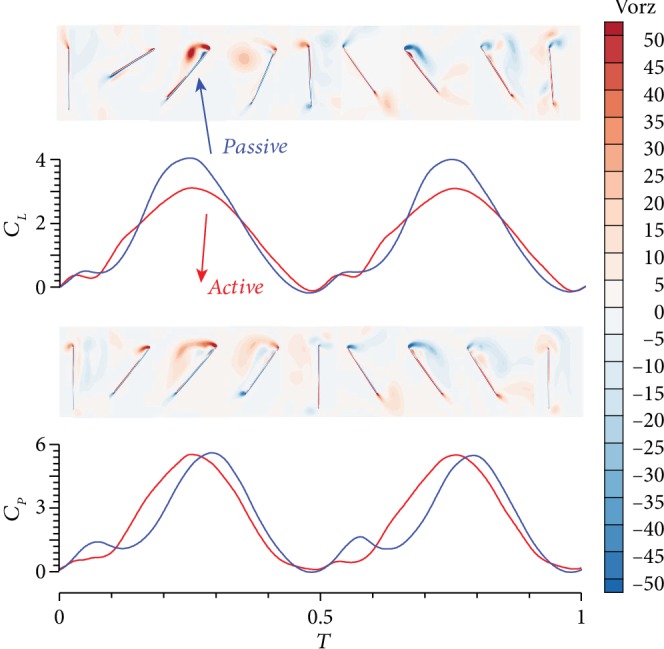
Comparison of *C*_*L*_, *C*_*P*_, and flow fields during upstroke between two models.

Based on the previous analysis, a conclusion can be drawn that the passive pitching wing can maintain a high aerodynamic efficiency while generating more lift, which is beneficial to FWMAVs to enhance the payload and implement the maneuver flight. Although a small loss of lift is observed at the beginning and the end of the upstroke/downstroke, the instantaneous lift at the middle stage significantly increases by nearly 30% (Figure 9) and the average lift in one cycle improves by 10%, with the coefficient changes from 1.519 to 1.671. For instantaneous power, the passive pitching wing consumes much more power in the initial phase of the upstroke/downstroke but greatly saves power in the phase of rotation. Overall, the average aerodynamic power consumption slightly differs between the active and passive pitching wings in one cycle; their coefficients are 2.287 and 2.291, respectively.

**Figure 10:**
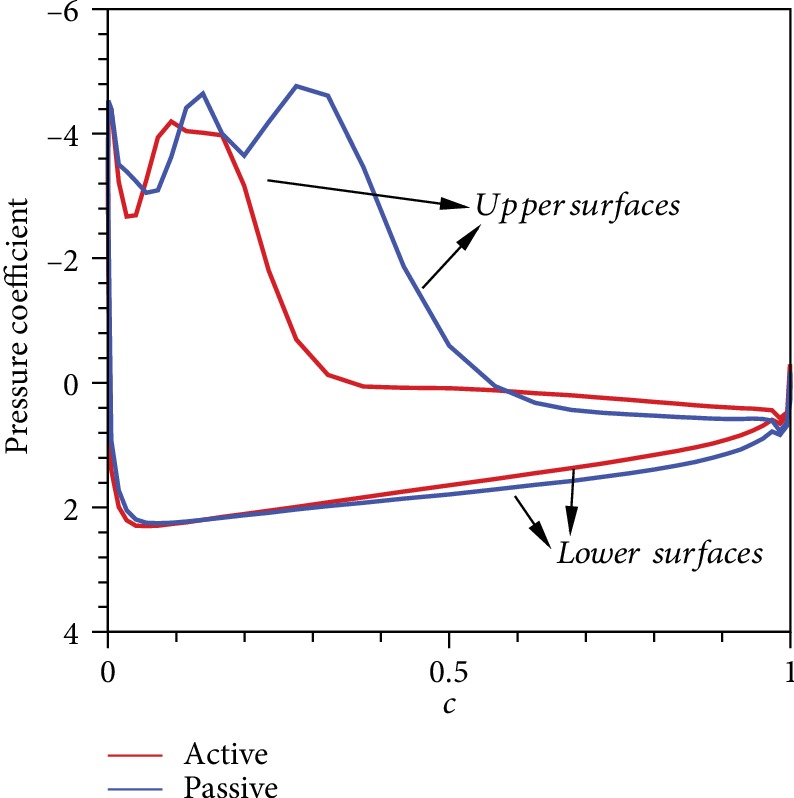
Comparison of the pressure distributions of the wing cross section at $\bar{R_{2}}$position in the middle of the upstroke.

Several differences can be observed in the flow field around the wings in the two models. The periodic motion causes LEV to develop and then decline. Subsequently, the LEV in the opposite direction begins to expand. During the entire process, the LEV attached to the wing surface ensures the distribution of aerodynamic forces. Figure 9 shows that no evident vorticity is observed around the flapping wing during the initial stage of the upstroke, and the generated lift is small. The LEV of the two models becomes increasingly significant as time progresses. However, the intensity of the active pitching model rapidly increases, and the lift is larger than that of the passive pitching model during the initial period. Subsequently, the LEV of the passive pitching model develops rapidly. A clear enhancement in lift is observed because vorticity is concentrated, attached to the surface, and continuous. This condition can also be explained by pressure distribution.  Figure 10 shows that the pressure difference between the upper and lower surfaces of the passive pitching wing is more considerable than that of the active pitching wing. LEV gradually sheds at the end of upstroke, and the lift declines. During this process, the vorticity of the passive pitching model remains relatively concentrated, whereas the vorticity of the active pitching model becomes dispersed.

We associate the aerodynamic force with vorticity in the flow field and attempt to explain the aforementioned phenomenon from another perspective. In an incompressible viscous flow, the relationship between aerodynamic force and vorticity is defined as [25]

(14) $$\begin{array}{c} \gamma_{f,b}^{*}=\int_{V_{f}+V_{b}}r^{*}\times \omega^{*}dV, \end{array}$$

where **ω**^∗^ is vorticity; **r**^∗^ is the position vector; *V*_*f*_ and *V*_*b*_ are the volumes of fluid and solid, respectively; and **γ**_**f**,**b**_^∗^ is the first moment of vorticity.

The aerodynamic force vector **F**^∗^ can be written as

(15) $$\begin{array}{c} F^{*}=-\frac{1}{2}\rho \frac{d\gamma_{f,b}^{*}}{{dt}^{*}}+\rho \frac{d}{{dt}^{*}}\int_{V_{b}}v^{*}dV, \end{array}$$

where **v**^∗^ represents the speed of a certain point in *V*_*b*_. Its dimensionless form is expressed as

(16) $$\begin{array}{c} F=-\frac{d\gamma_{f,b}}{d\tau }+\frac{2}{\rho c}\frac{d}{d\tau }\int_{V_{b}}vdV, \end{array}$$

where **F** = 2**F**^∗^/*ρU*^2^*S*, **γ**_**f**,**b**_ = **γ**_**f**,**b**_^∗^/*UcS*, and **v** = **v**^∗^/*U*.

If the wing rotates at a constant speed, then the first term at the right of equation (16) can be written as $-4{\dot{\varphi }}^{2}\left(V_{b}/Sc\right)\left(r_{m}/c\right)$, where *r*_*m*_ is the position of the wing centroid, and the second term at the right of equation (16) can be written as $-2{\dot{\varphi }}^{2}\left(V_{b}/Sc\right)\left(r_{m}/c\right)$. *V*_*b*_/*Sc* is small when the wing is thin. Thus, the two terms are small. Equation (16) can be approximated as

(17) $$\begin{array}{c} F=-\frac{d\gamma }{d\tau }, \end{array}$$

where **γ** is the sum of the first moments of vorticity in the fluid. The lift and drag coefficients can be written as

(18) $$\begin{array}{c} C_{L}=\frac{d\left(-\gamma_{y}\right)}{d\tau },\\ C_{D}=\frac{d\gamma_{x}}{d\tau }\cos\varphi +\frac{d\gamma_{z}}{d\tau }\sin\varphi , \end{array}$$

where *γ*_*x*_, *γ*_*y*_, and *γ*_*z*_ are the components of **γ** in the *x*, *y*, and *z* directions, respectively.

**Figure 11:**
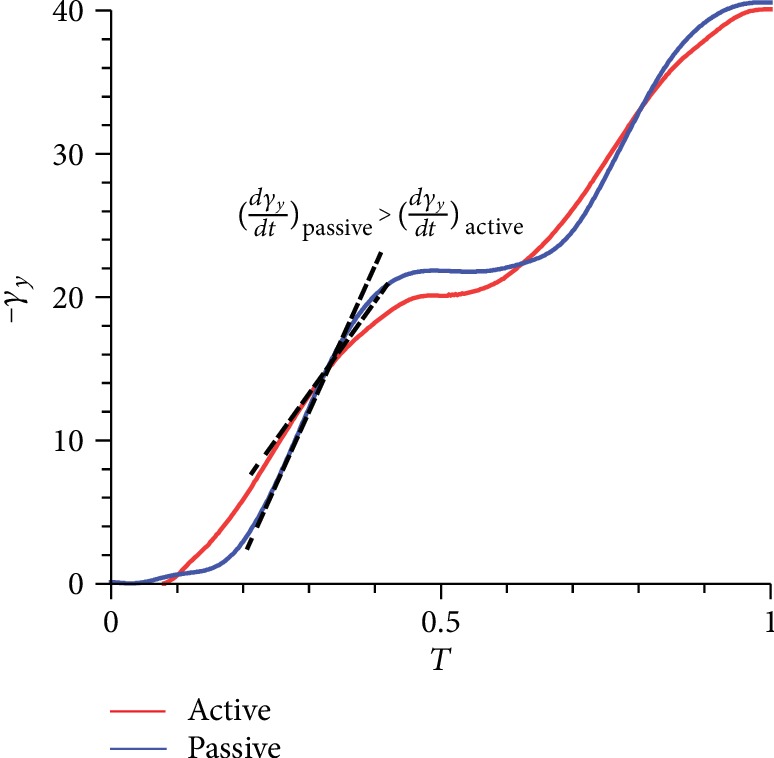
Comparison of the first moment of vorticity in one cycle between the two models.

Equation (18) indicates that aerodynamic force is proportional to the time rate of change in the first moment of vorticity. Since the *γ*_*y*_ curve of passive pitching has a larger slope in the middle of the upstroke/downstroke (*T* ≈ 0.2–0.4/*T* ≈ 0.7–0.9) than that of active pitching (Figure 11), the lift of the passive pitching wing is greater than that of the active pitching wing during this period. In combination with the characteristic of *α* (Figure 5), we assume that the rapid change in vorticity may be attributed to the second small peak, indicating the occurrence of a sudden reverse pitch motion.

### 3.4. Control Strategies in the Passive Pitching Model

Despite of a higher lift compared to active pitching wing, the passive wing kinematic modulations are energetically efficient [9]. Early studies on fruit flies have drawn conclusions from various observations and experiments that fruit flies asymmetrically change the twist angle of their left and right wings and drive their body to complete a lateral movement [22]. Given that the passive pitching model is based on the characteristic of insects, we infer that a similar effect can be achieved in the design of FWMAV [3].

In our calculation, the flapping wing is in an equilibrium position when *α* = 90°. At this time, the torsional spring exhibits no angular displacement and the recovery moment is 0. In the previous analysis, *α*_0_ = 90° and the initial position of the wing is the equilibrium position. However, the initial position of the wing deviates from the equilibrium position when *α*_0_ ≠ 90°. The symmetry of *α* during the upstroke and downstroke is broken, thereby increasing horizontal and vertical forces and resulting in a moment around the wing root. Almost no lift loss is observed when a moment is produced.

**Figure 12:**
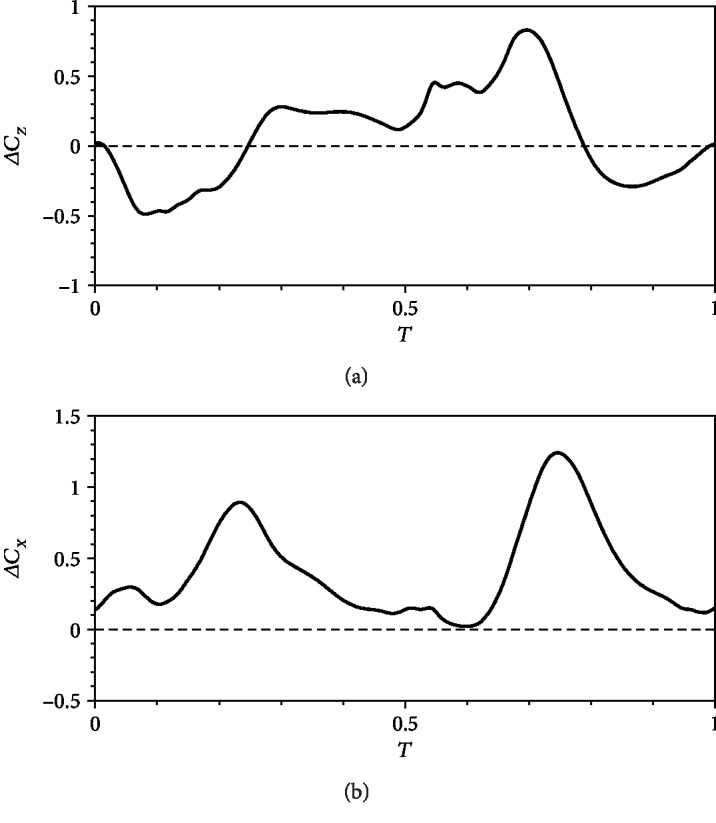
Differences in (a) horizontal force coefficient and (b) vertical force coefficient between *α*_0_ = 70° and *α*_0_ = 90°.

Figure 12 shows that relative speed and drag increase during the upstroke as *α*_0_ decreases, thereby causing a positive variation in horizontal force. During downstroke, relative speed and drag decrease, thereby causing a positive variation in horizontal force. Thus, a large yaw moment is generated around the wing root. Simultaneously, the lift increases during upstroke, thereby increasing the vertical force. Then, the lift decreases during downstroke, consequently decreasing the vertical force. As such, the variations in vertical forces during the upstroke and downstroke cancel each other. However, their distribution contributes to the pitch moment around the wing root.

**Figure 13:**
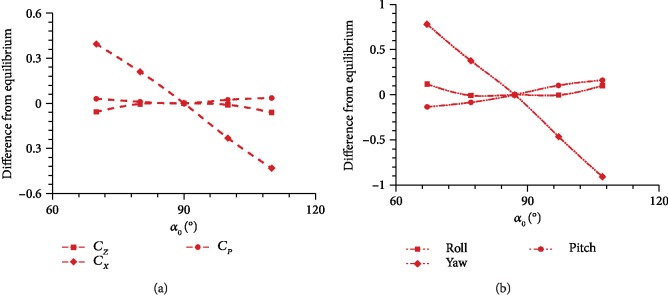
(a) Horizontal force, vertical force, and power coefficient at different *α*_0_ and (b) roll, yaw, and pitch moments at different *α*_0_.

In Figure 13, the average aerodynamic power almost remains the same when *α*_0_ changes from 70° to 110°. The rest angle of the torsional spring can be used as a control variable in applying the passive pitching model. The adjustment of *α*_0_ on the left and right wings controls the attitude and trajectory of the aircraft during flight. This process requires neither complex auxiliary mechanisms nor additional power input, and this characteristic is an advantage that is not exhibited by the active pitching model.

## 4. Conclusions

We investigate the aerodynamic performance of the passive pitching model on FWMAVs via 3D numerical simulation and demonstrate that the angle of attack exhibits the characteristic of “double peak oscillation” under the combination of aerodynamic, spring, and inertial moments in the simplified passive pitching model, which simulates the motion of insect wings well. Torsional stiffness considerably affects aerodynamic force and efficiency in the passive pitching model. Excess rigidity or flexibility deteriorates the performance. According to the comparison between active and passive pitching wings, with appropriate torsional stiffness, the average lift can be enhanced by 10% at the same aerodynamic efficiency when the wing pitches passively. Simultaneously, the yaw moment around the wing root can be obtained to assist the control system without losing lift by setting different rest angles for the left and right wings. These results show that the passive pitching model positively contributes to the improvement of the hovering and maneuverability of FWMAVs. In the future, we will conduct a series of studies about the effect on the stability caused by passive pitching wing to further investigate this bionic model.

## Figures and Tables

**Table 1:** *α*, $\bar{C_{L}}$, $\bar{C_{P}}$, and *η* corresponding to different *k*.

*k*	Max/min *α*	$\bar{C_{L}}$	$\bar{C_{P}}$	*η*
6.4	105°/74°	1.196	7.394	0.177
1.2	132°/48°	2.109	4.476	0.684
0.15	165°/9°	0.481	1.033	0.323

## Data Availability

The data used to support the findings of this study are included within the article. The detailed calculation results are available from the corresponding author upon request. The program and source code have not been made available because of privacy protection.
